# A Brief Sketch of a Few Extraordinary Surgical Cases

**Published:** 1871-10

**Authors:** E. R. Willard

**Affiliations:** Wilmington, Illinois


### A BRIEF SKETCH OF A FEW EXTRAORDINARY SURGICAL CASES.

By E. R. WILLARD, M. D., Wilmington, Illinois.

Case I. Mr. B., a short, robust man, of motive temperament, about 40 years of age, employed at the Wilmington coal mines to work in the "Y" shaft, while somewhat intoxicated on the evening of September 20th, 1868, accidentally walked into the shaft at the top of the ground. As the cage was at the bottom at the time, a distance of some sixty-eight feet, and the sides of the shaft as smooth as a floor, one would suppose that a man making such a leap and striking upon a solid platform, would be instantly killed; but such was not tne case. He was immediately discovered, in an insensible condition, and taken out of the shaft to his boarding-house. The attending physician, Dr. Denormandy, was instantly sent for, and apprehending from the severity of the injuries that it would be necessary to amputate one, if not both legs, I was called in. Found the man partially insensible, and both legs broken. The astragalus of the right foot thrust up between the tibia and fibula, with fracture of the latter bones, about five inches from the ankle joint; while the tibia and fibula of the leg were broken about three inches from the joint, and the ankle dislocated outwards, with solution of continuity of the external malleolus. There was pulsation in both anterior and posterior tibial arteries, which induced me to recommend an attempt to save the legs, knowing that amputation could be performed at any subsequent time, should it be found necessary. With that understanding, so soon as the necessary preparations could be made, we reduced the fractures and dislocations after much careful manipulation, and dressed with side-splints and fracture-boxes. To relieve the extreme collapse and allay pain, carbonate of ammonia was freely given, together with small doses of morphine.

From this the patient rapidly recovered, and in three months was upon his feet again.

---

CASE II. Upon the 5th day of October, 1869, four men stepped upon the cage at the top of the shaft at the Gardner coal mines, for the purpose of descending into the pit, a distance of one hundred and ninety-five feet. As the engineer started the machinery for lowering the cage, the rope broke, precipitating the cage and men to the bottom, instantly killing one man, producing compound comminuted fracture of the leg of the second, slightly injuring the spine of the third, while the fourth received a severe blow upon the lumbar vertebræ, causing paralysis of the lower extremities. Pins thrust into the skin at any point below the seat of injury, gave not the slightest sensation of pain.

The attending physician, Dr. McMann, of Gardner, thinking the case a capital one to try "Clines's Operation" of relieving the compression of the spinal cord by cutting down upon the injured part, and sawing through the lamina sufficiently to elevate the depressed portion of bone, immediately telegraphed for me to meet him in consultation. Upon arriving I learned the above facts, and after a careful survey of the case, notwithstanding the want of success hitherto attending the operation, I advised its immediate performance. So soon as the necessary arrangements were completed, we made an incision about six inches in length, through the integument over the seat of injury, and separated the muscles upon either side of the spinous processes sufficiently to ascertain the extent of injury. This done, we discovered the second lumbar vertebræ fractured through the lamina, and the spinous process driven in upon the cord, and so wedged as to be perfectly immoveable with the tooth forceps. Hayes's saw was applied, and the lamina divided upon either side, and the process elevated, when the spinal cord was found to be so completely contused and lacerated as to entirely obliterate its functional activity.

The wound was properly dressed, the water drawn as occasion required, and the bowels attended to whenever necessary. From this the patient lived about ten days, and died.

---

Case III. May 7th, 1870, M. K——, a stout, robust man of middle age, while working in the Gardner Coal Mines, was struck upon the back by a large stone falling from the roof, weighing some four hundred pounds. He was immediately taken from the shaft and Dr. McMann called in. After a careful examination of the case he diagnosed fracture of the third lumbar veterbræ with contusion of the spinal cord. For which Cline's operation was advised. With this view I was summoned. Upon arriving I found the case as above stated, with complete loss of both sensation and motion of the lower extremities, together with paralysis of the bladder. Although the injury seemed to be of so severe a nature as to almost entirely preclude any prospect of success in an operation; still I thought best to operate knowing that death would soon follow without the operation, should the cord prove to be seriously injured; also that the danger of the patient could not thereby be increased.

After the patient and friends were made thoroughly acquainted with the nature of the operation, we proceeded as in the former case. Upon incising the integuments we found the yellow ligaments connecting the lamina of the two vertebra torn through, the cord completely separated, and the spinous process of the third lumbar vertebræ drove down upon the body of the same, thus precluding all possible chance of recovery. This man lived some thirteen days after the operation.

*Remarks.*—Although this operation for the removal of the pressing vertebræ arch, was first proposed by Henry Cline in 1814, some fifty-seven years ago, still no favorable results have as yet attended its performance. This I attribute to the fact that the cases were extremely unfavorable for the operation, except Tyrells, and one or two others. In the above cases the sheath and cord were so completely torn through in both instances that the operation at once showed the utter impossibility of a favorable issue in either case. That the operation hastened in the least the death of either of the patients, I have my doubts, and would be tempted to operate again under similar circumstances.

Were it possible for us to ascertain whether the cord was simply compressed, or entirely torn through, or whether the symptoms resulted from blood effusions in the different situations before the operation, then we think its performance would be as certain of success as trephining in fracture of the skull with compression.

CASE IV. Before day-light on the morning of December 29th, 1870, Mr. C——, the engineer at the R shaft left the engine-room for the purpose of oiling some of the machinery. When near the shaft his lantern "blew out," and supposing himself some distance from the opening, continued to walk forward until he stepped into the shaft. The cage being at the bottom at the time, he fell astride the crane, some sixty feet below, severely lascerating the perineum and urethra, besides producing severe and extreme contusion of the testicles and soft parts about the nates and anus. The attending physician, Dr. Payne, saw the patient soon after the accident, and immediately made cold applications to the parts. In a few hours he was again summoned for the purpose of relieving the bladder, as it was found the patient could not micturate. After making several ineffectual attempts to pass the catheter, I was called in consultation. Upon arriving, I found the parts so contused, echymcsed, and infiltrated with urine, together with the edemutous swelling, as to have the "feel" and appearance of jelly. The bladder was also found distended to its utmost capacity. This I attempted to relieve by a medium sized catheter; failing in this, I resorted to a number six, and after introducing it down to the point of injury in the uretha, I carefully enlarged the opening in the integuments, dissecting down to the urethra sufficient to introduce the fingers, by which, after long and careful manipulation I succeeded in introducing the catheter. This was made fast and retained for a number of weeks. Meantime the bladder was frequently syringed out with luke-warm water, water dressings continued to the parts, and anodynes given whenever necessary to relieve pains and procure sleep. From this the patient rapidly improved, and the extensive sloughing was soon replaced by healthy granulations.

[TO BE CONTINUED.]

